# Metabolic dysfunction-associated fatty liver disease increases risk of adverse outcomes in patients with chronic hepatitis B

**DOI:** 10.1016/j.jhepr.2021.100350

**Published:** 2021-08-08

**Authors:** Laurens A. van Kleef, Hannah S.J. Choi, Willem P. Brouwer, Bettina E. Hansen, Keyur Patel, Robert A. de Man, Harry L.A. Janssen, Robert J. de Knegt, Milan J. Sonneveld

**Affiliations:** 1 Department of Gastroenterology and Hepatology, Erasmus MC University Medical Centre, Rotterdam, The Netherlands; 2 Toronto Centre for Liver Disease, University Health Network, Toronto, Canada

**Keywords:** Chronic hepatitis B, CHB, Hepatitis B, HBV, Metabolic dysfunction-associated fatty liver disease, MAFLD, Adverse clinical outcomes, Survival, Hepatocellular carcinoma, HCC, Steatohepatitis, aHR, adjusted hazard rate, ALT, alanine aminotransferase, CHB, chronic hepatitis B, FLD, fatty liver disease, HCC, hepatocellular carcinoma, HR, hazard rate, MAFLD, metabolic dysfunction-associated fatty liver disease, NAFLD, non-alcoholic fatty liver disease, NAS, NAFLD activity score, NASH, non-alcoholic steatohepatitis, NHANES, National Health and Nutrition Examination Survey, P25–P75, 25th–75th percentile, ULN, upper limit of normal

## Abstract

**Background & Aims:**

A recent consensus document has defined metabolic dysfunction-associated fatty liver disease (MAFLD) as hepatic steatosis together with overweight, diabetes, and/or a combination of other metabolic risk factors. The clinical relevance of this novel diagnosis is unknown among patients with chronic hepatitis B (CHB). We studied the association between MAFLD (with or without steatohepatitis) and adverse clinical outcomes in patients with CHB.

**Methods:**

We performed a retrospective long-term follow-up cohort study at 2 tertiary hospitals in patients with CHB who underwent liver biopsy. Biopsies were reassessed for steatosis, degree of fibrosis, and presence of steatohepatitis. Associations with event-free hepatocellular carcinoma (HCC)-free and transplant-free survival were explored.

**Results:**

In our cohort, 1076 patients were included, median follow-up was 9.8 years (25th–75th percentile: 6.6−14.0), and 107 events occurred in 78 patients, comprising death (n = 43), HCC (n = 36), liver decompensation (n = 21), and/or liver transplantation (n = 7). MAFLD was present in 296 (27.5%) patients and was associated with reduced event-free (adjusted hazard ratio [aHR] 2.00, 95% CI 1.26–3.19), HCC-free (aHR 1.93, 95% CI 1.17–3.21), and transplant-free survival (aHR 1.80, 95% CI 0.98–3.29) in multivariable analysis. Among patients with MAFLD, the presence of steatohepatitis (*p* = 0.95, log-rank test) was not associated with adverse outcomes.

**Conclusions:**

The presence of MAFLD in patients with CHB was associated with an increased risk for liver-related clinical events and death. Among patients with MAFLD, steatohepatitis did not increase the risk of adverse outcomes. Our findings highlight the importance of metabolic dysfunction in patients with CHB.

**Lay summary:**

Recently, metabolic dysfunction-associated fatty liver disease (MAFLD) has been defined as fatty liver disease with signs of metabolic dysfunction. Among patients with chronic hepatitis B, MAFLD was associated with liver-related events and death. Metabolic health assessment should be encouraged among patients with chronic hepatitis B, especially in those with fatty liver disease.

## Highlights

• Recently, MAFLD was defined as hepatic steatosis in the presence of metabolic dysfunction (*e.g.* overweight, diabetes).
• In patients with chronic hepatitis, MAFLD was associated with reduced event-free (*p* <0.001), HCC-free (*p* <0.001), and transplant-free survival (*p* <0.001).
• Among patients with MAFLD, risk of adverse outcomes was similar for patients with and patients without steatohepatitis.
• Our findings underscore the importance of metabolic dysfunction in patients with CHB.

## Graphical abstract

**Figure:**
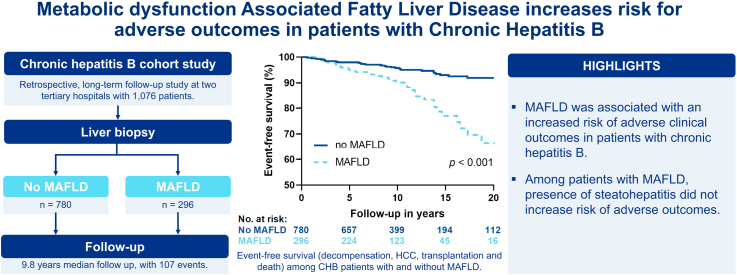

## Introduction

Chronic hepatitis B (CHB) is the most common form of chronic viral hepatitis, with an estimated 3.9% prevalence globally.^1^ As a consequence of liver cirrhosis and hepatocellular carcinoma (HCC), CHB results in an estimated 887,000 deaths annually.^2^ The available antiviral agents effectively suppress HBV DNA and reduce but not eliminate the risk of adverse clinical outcomes.^3^ The persistent risk of adverse outcomes may be partially attributable to the presence of co-existing liver diseases such as fatty liver disease. Chronic hepatitis B infection is very much endemic in the Asia-Pacific region, where the prevalence of non-alcoholic fatty liver disease (NAFLD) has also increased rapidly over the last decennia and now matches or even exceeds prevalence in Europe.[4], [5], [6] NAFLD is expected to become the leading cause of liver-related morbidity and the main indication for liver transplantation globally^4^^,^^7^ and already accounts for 21.5% of the transplantations in the United States.^8^

With the prevalence of NAFLD rapidly increasing in the regions where HBV infection is most common, a large population is potentially at risk for having 2 concomitant liver diseases, which may result in a synergistic effect on the risk of HCC, cirrhosis, and death. Indeed, (severe) steatosis has been linked to more advanced liver fibrosis and a higher risk of HCC in patients with CHB.[9], [10], [11] A previous study from our group also suggested that the presence of non-alcoholic steatohepatitis (NASH) was an important driver of this association.^12^ However, assessment of the role of NAFLD in patients with CHB is complex because it requires exclusion of secondary causes of steatosis (one of which may be HBV infection). Moreover, the assessment of steatohepatitis may be challenging in patients with alternative causes of liver inflammation such as active viral hepatitis.

Recently, a transition from NAFLD to metabolic dysfunction-associated fatty liver disease (MAFLD) was introduced at an international expert consensus meeting.^13^ Hence, diagnosis is no longer based on exclusion of secondary causes for steatosis and/or presence of steatohepatitis, but the focus has shifted towards positive diagnostic criteria based on a combination of significant hepatic steatosis and presence of (components of) the metabolic syndrome. Although this rather practical approach underlines the importance of metabolic dysfunction in the pathogenesis of fatty liver disease, the clinical relevance of this novel classification is yet unknown. In the National Health and Nutrition Examination Survey (NHANES) III cohort, the novel definition of MAFLD yielded a similar prevalence when compared with the conventional NAFLD criteria,^14^ and similar observations were made in cohorts from Hong Kong and Japan.^15^^,^^16^ Moreover, among patients with MAFLD, CHB was associated with more inflammation and fibrosis.^17^ However, the impact of superimposed MAFLD on long-term clinical outcomes, and the clinical significance of the concomitant presence of biopsy-proven steatohepatitis, is still unclear.

Therefore, in this study, we aimed to investigate the association between MAFLD (with or without steatohepatitis) and adverse clinical outcomes in patients with CHB.

## Patients and methods

### Patients

This was a multicentre retrospective cohort study comprising all HBsAg-positive patients who underwent liver biopsy between 2005 and 2016 at the Toronto Centre for Liver Disease in Toronto, Canada, and between 1985 and 2012 at the Erasmus University Medical Centre in Rotterdam, The Netherlands.^12^ Baseline assessment was set at date of liver biopsy.

### Biopsy assessment

All liver biopsies were reassessed by 3 dedicated, experienced, tertiary centre histopathologists for the presence of steatosis (positive if >5%, according to the Brunt classification),^18^ degree of fibrosis (based on METAVIR score),^19^ inflammatory activity,^20^ and presence of ballooning. Steatohepatitis was defined as the combined presence of steatosis, inflammatory activity, and ballooning. Moreover, for sensitivity analysis, steatohepatitis was based on NAFLD activity score (NAS) ≥3,^21^ as used previously.^12^

### Follow-up and endpoints

Data regarding antiviral treatment and events, defined as HCC, liver decompensation, liver transplantation, and all-cause mortality, were collected from the electronic medical records or local registries through February 2018. The primary endpoint for this study was event-free survival, with liver transplant-free survival and HCC-free survival assessed as secondary endpoints.

### Patient classification

Patients with >5% steatosis or steatohepatitis on liver biopsy were classified as having fatty liver disease. Patients with fatty liver disease were classified as MAFLD in the presence of either a BMI ≥25 kg/m^2^ (non-Asians) or ≥23 kg/m^2^ (Asians) or diabetes mellitus. Thereafter, we re-assessed the charts of nonoverweight patients without diabetes but with fatty liver disease for the presence of ≥2 minor metabolic health comorbidities (such as hypertension and dyslipidaemia).^13^ Patients with fatty liver disease without sufficient data for assessment of MAFLD (defined as missing data on BMI in the absence of other MALFD criteria) were excluded (n = 13). Patients within the MAFLD group were further classified as MAFLD with and MAFLD without steatohepatitis based on the biopsy results.

### Statistical analysis

Cohort characteristics were described with normally distributed variables presented as mean, SD, and non-normally distributed variables as median ±25th–75th percentile (P25–P75). Distribution was assessed visually and by skewness and kurtosis. ANOVA was used to study differences for normally distributed continuous data, the Kruskal–Wallis test for non-normally distributed continuous data, and the Chi-square test for categorical data. Kaplan–Meier analysis with log-rank statistics was used for survival analysis. Cox proportional hazards models were applied for multivariable analysis. Multivariable models were adjusted for age, sex, HBeAg serostatus, advanced fibrosis, and antiviral treatment based on factors previously identified as predictors of adverse outcomes in this cohort.^12^ All analyses were performed in R 4.0.3 with the Survival package 3.2-3. A *p* value of <0.05 was considered statistically significant.

### Ethics

This study was conducted according to the principles set forth in the Declaration of Helsinki. The requirement for informed consent was waived, and the individual institutional review boards gave necessary approval. All authors had access to the study data and reviewed and approved the final manuscript.

## Results

### Patient characteristics

**Fig. 1:**
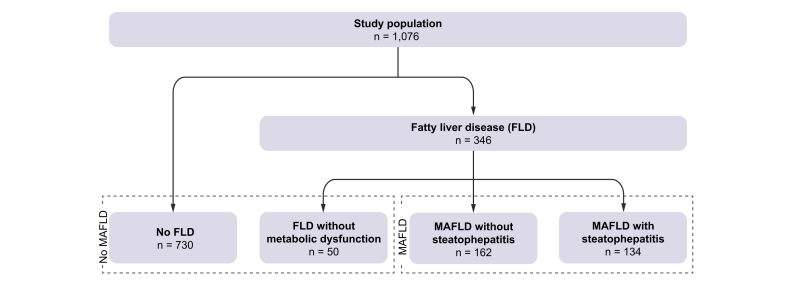
Flowchart study population. FLD, fatty liver disease; MAFLD, metabolic dysfunction-associated fatty liver disease.

This cohort comprised 1089 patients, of whom 13 patients were excluded because of insufficient data for MAFLD classification. Among the remaining 1076 patients, fatty liver disease was detected in 346 (32%), of whom 296 (86%) had MAFLD (Fig. 1). MAFLD diagnosis was predominantly (96.3%) based on the presence of steatosis with overweight and/or diabetes. Among patients with MAFLD, 134/296 (45%) had steatohepatitis and 156/296 (53%) had NAS ≥3.

At study enrolment, the median age was 38.6 years, 66% were male, and the majority (57%) had Asian ethnicity. In the overall cohort, 52% were overweight/obese, 5% had diabetes, and 11% had hypertension and/or hyperlipidaemia. The majority of patients had elevated alanine aminotransferase (ALT) (70%) at the time of biopsy. Patients with MAFLD were significantly older, were more frequently male, and more often had advanced fibrosis (Table 1). Characteristics for MAFLD with or without steatohepatitis are shown in Table S1.

**Table 1:** **Patient characteristics**.

Variable	No MAFLD (n = 780)	MAFLD (n = 296)	*p* value
Age (years)	36.7 (13.2)	43.6 (11.7)	<0.001
Female, n (%)	310 (39.7)	59 (19.9)	<0.001
Race, n (%)			0.573
Caucasian	216 (27.7)	92 (31.1)	
Asian	448 (57.4)	168 (56.8)	
African/Black	96 (12.3)	30 (10.1)	
Other	20 (2.6)	6 (2.0)	
Overweight,∗ n (%)	279 (35.8)	278 (93.9)	<0.001
Hypertension/hyperlipidaemia, n (%)	47 (6.0)	76 (25.7)	<0.001
Diabetes, n (%)	20 (2.6)	34 (11.5)	<0.001
ALT (lU/L)	52 [33, 95]	53 [38, 80]	0.409
Elevated ALT,† n (%)	512 (68.5)	216 (75.8)	0.027
HBeAg positive, n (%)	385 (49.5)	91 (30.7)	<0.001
HBV DNA (log IU/ml)	5.71 (2.63)	4.82 (2.64)	<0.001
Hepatic activity (A2–A4), n (%)	394 (50.5)	153 (51.7)	0.782
Advanced fibrosis (F3–F4), n (%)	197 (25.3)	94 (31.8)	0.041

Data are presented as mean (SD), median [P25–P75], or n (%). ANOVA was used to study differences for normally distributed continuous data, the Kruskal–Wallis test for non-normally distributed continuous data, and the Chi-square test for categorical data.

ALT, alanine aminotransferase; MAFLD, metabolic dysfunction associated fatty liver disease; ULN, upper limit of normal.

∗ BMI >25 kg/m^2^ (non-Asians) or >23 kg/m^2^ (Asians).

† Exceeding local ULN.

Median follow-up was 9.8 years (P25–P75: 6.6–14.0), resulting in 11.729 person-years of follow-up. Overall, 107 events occurred in 78 patients, comprising death (n = 43), HCC (n = 36), liver decompensation (n = 21), and/or liver transplantation (n = 7). The number of events per group is shown in Table S2.

### MAFLD is associated with impaired event-free and HCC-free survival

**Fig. 2:**
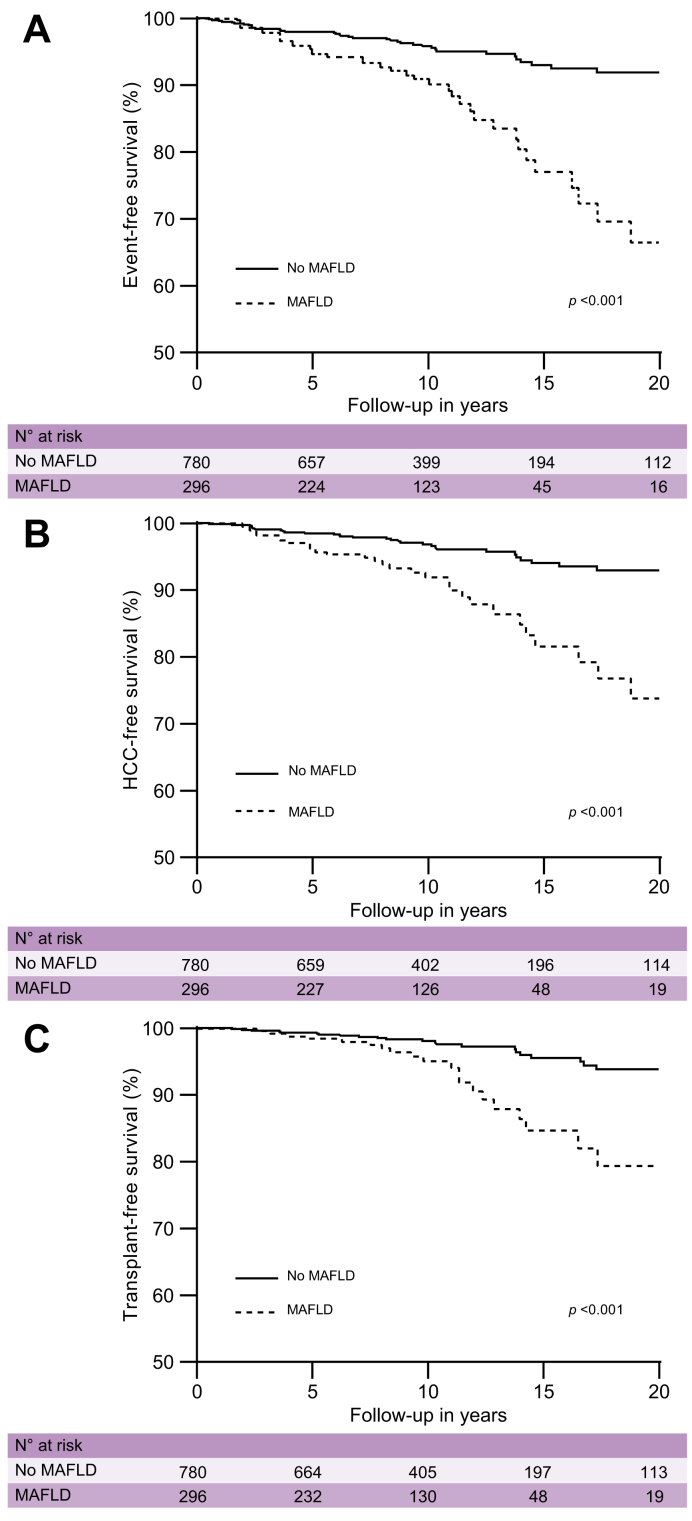
Association of MAFLD with (A) event-, (B) HCC-, and (C) transplant-free survival. Results were obtained with Kaplan–Meier analysis with log-rank statistics. HCC, hepatocellular carcinoma; MAFLD, metabolic dysfunction-associated fatty liver disease.

**Fig. 3:**
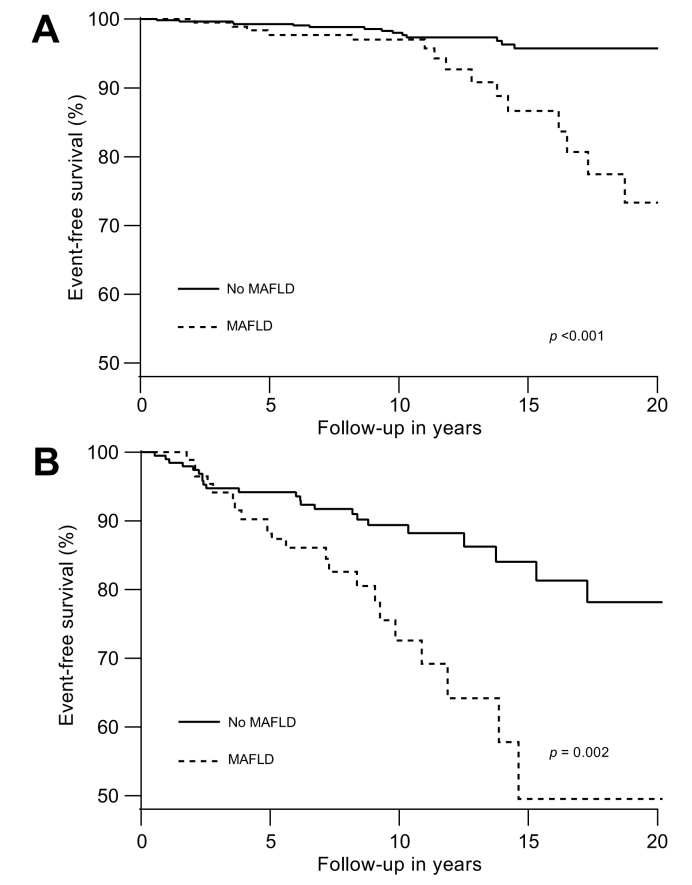
Association of MAFLD for event-free survival in (A) patients without and (B) with advanced fibrosis. Results were obtained with Kaplan–Meier analysis with log-rank statistics. Advanced fibrosis: METAVIR F3–F4. MAFLD, metabolic dysfunction-associated fatty liver disease.

The presence of MAFLD was associated with a significantly decreased event-free survival in the overall population (*p* <0.001, Fig. 2A), which was consistent in patients with (Fig. 3A) and without (Fig. 3B) advanced fibrosis at study enrolment. Additionally, MAFLD was associated with reduced HCC-free survival (*p* <0.001, Fig. 2B) and transplant-free survival (*p* <0.001, Fig. 2C).

Similar results were obtained in multivariate analysis (Table 2), where MAFLD was independently associated with a reduced event-free survival (adjusted hazard rate [aHR] 2.00, 95% CI 1.26–3.19), HCC-free survival (aHR 1.93, 95% CI 1.17–3.21), and transplant-free survival (aHR 1.80, 95% CI 0.98–3.29), adjusted for age, sex, HBeAg serostatus, advanced fibrosis, and antiviral treatment. Additional adjusting for ALT, ethnicity, or medical centre did not result in significant changes of the described associations. Findings were consistent when only liver-related outcomes were assessed: MAFLD increased the risk of incident HCC (aHR 1.96, 95% CI 1.00–3.86, *p* = 0.049) and of a composite endpoint comprising only liver-related events (decompensation, HCC, or liver transplant; aHR 2.19, 95% CI 1.26–3.83, *p* = 0.006).

**Table 2:** **MAFLD and adverse outcomes**.

Outcome	HR	95% CI	*p* value
Clinical event∗			
Unadjusted	3.01	1.91–4.73	<0.001
Multivariate†	2.00	1.26–3.19	0.003
HCC/transplant/death			
Unadjusted	3.04	1.85–4.98	<0.001
Multivariate†	1.93	1.17–3.21	0.011
Transplant/death			
Unadjusted	2.82	1.56–5.09	<0.001
Multivariate†	1.80	0.98–3.29	0.058

Results were obtained with Cox proportional hazards analysis and given as HR with 95% CI.

HCC, hepatocellular carcinoma; HR, hazard ratio.

∗ Clinical event: decompensation, HCC, transplant, or death.

† Adjusted for age, sex, HBeAg serostatus, advanced fibrosis, and antiviral treatment.

### Similar outcomes in patients with MAFLD with or without steatohepatitis

**Fig. 4:**
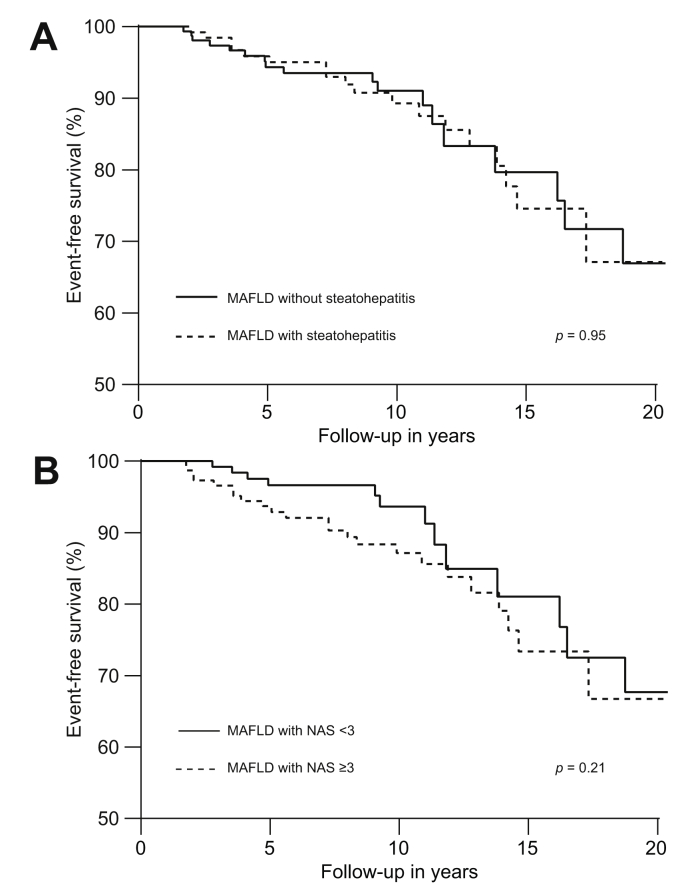
Event-free survival in patients with MAFLD according to presence of (A) steatohepatitis and (B) NAS ≥3. Results were obtained with Kaplan–Meier analysis with log-rank statistics. MAFLD, metabolic dysfunction-associated fatty liver disease; NAFLD, non-alcoholic fatty liver disease; NAS, NAFLD activity score.

Event-free survival was similar for patients with MAFLD irrespective of the presence of steatohepatitis (*p* = 0.95, Fig. 4A) or the presence of NAS ≥3 (*p* = 0.21, Fig. 4B). These results were consistent in multivariable analysis: no associations with adverse outcomes were found for steatohepatitis (*p* = 0.91) and NAS ≥3 (*p* = 0.38) among patients with MAFLD.

### Fatty liver disease without metabolic dysfunction is not associated with adverse outcomes

Among 346 patients with fatty liver disease, 50 had no metabolic risk factors and therefore did not comply with the MAFLD criteria. Event-free survival was similar in these patients compared with patients without signs of fatty liver disease (*p* = 0.56, Fig. 1). Similar results were obtained in multivariate analysis (aHR 0.77, 95% CI 0.26–2.29).

## Discussion

In this large multi-ethnic multicentre cohort study, the presence of MAFLD was independently associated with impaired event-free, HCC-free, and transplant-free survival in patients with CHB. Among patients with MAFLD, concomitant presence of steatohepatitis did not increase the risk of adverse outcomes.

Various recent studies have shown that the obesity pandemic has spread outside the Western World to regions endemic for hepatitis B, leading to an increased prevalence of fatty liver disease in the population with CHB.[4], [5], [6] The clinical relevance of concomitant steatosis in patients with CHB has long been debated, as several studies, including a meta-analysis, could not identify steatosis as a risk factor for adverse outcomes.^22^ One of the reasons for these contradictory results could be the steatogenic effect of HBV infection. Several potential molecular pathways leading to steatosis are identified for hepatitis B.[23], [24], [25] Interestingly, patients with CHB and steatosis may also have up to 3 times increased rates of HBsAg clearance.^9^ This complex interplay can result in underestimating the steatogenic effect of HBV and subsequently complicate research into the clinical relevance of fatty liver disease in patients with CHB.

Importantly, recent studies have shown higher rates of significant fibrosis in patients with CHB and steatosis.^26^ Our study confirms this association using biopsy-based fibrosis assessment: patients with MAFLD were significantly more likely to have advanced fibrosis at the time of study enrolment. Furthermore, a recent study from our group identified NASH as a risk factor for clinical events in patients with CHB and advanced fibrosis.^12^ An important limitation of using NASH as a predictor of adverse outcomes is the requirement for liver biopsy, which is invasive and associated with a risk of severe complications. Besides, the considerable interobserver variability reported in previous studies is a major concern.^27^ Furthermore, assessment of NASH may even be more complicated in patients with CHB, as many histopathological hallmarks of steatohepatitis may also be accounted for by the presence of concomitant HBV-associated inflammation.

Given the contrasting findings regarding the importance of steatosis and the major limitations of using biopsy-proven NASH for risk stratification in HBV, using the novel MAFLD criteria to identify patients at higher risk of adverse outcomes could be of major clinical relevance.

In our cohort, the vast majority of patients with fatty liver disease (86%) also complied with MAFLD criteria, predominantly as a result of overweight (94%). In our study, superimposed MAFLD was associated not only with a significantly impaired event-free, HCC-free, and transplant-free survival but also with incident HCC or liver-related events. This emphasises that impaired event-free survival in this study is driven not only by increased mortality, which may be partly attributable to cardiovascular disease, but also by increased risk of liver-related events. The identification of MAFLD as a risk factor for decreased event-free survival and increased risk of HCC is in line with previous studies showing that metabolic comorbidity (*e.g.* diabetes and metabolic syndrome) is a risk factor for adverse outcomes in patients with CHB.^11^^,^^28^^,^^29^ This raises the question of whether MAFLD, even in the absence of advanced fibrosis, is an indication for HCC surveillance. Additionally, future studies should assess whether MAFLD may be a contributing factor for the persistently elevated risk of HCC observed in patients otherwise adequately treated for CHB.^30^

Among the patients with MAFLD in our cohort, 45% had concomitant steatohepatitis. Importantly, steatohepatitis in these patients was not associated with impaired event-free survival, despite being an important predictor for adverse outcomes in a population not stratified for MAFLD.^12^ Results were consistent for concomitant presence of NAS ≥3. This indicates that the disease burden of fatty liver disease is not limited to patients with NASH but extends to patients with MAFLD without steatohepatitis. Moreover, these findings suggest that when using the novel MAFLD definition, liver biopsy may not be essential for prognostic assessment of steatohepatitis in patients with both MAFLD and CHB but could be replaced by thorough metabolic assessment.

Another interesting observation in our cohort is that 14% of patients with fatty liver disease did not comply with the MAFLD criteria. This might reflect either HBV-associated steatosis or so-called lean fatty liver disease. These patients were not at increased risk for adverse outcomes compared with patients with CHB without fatty liver disease. These findings further underscore the importance of metabolic dysfunction, rather than fatty liver disease itself, with adverse outcomes.

Given the importance of metabolic health in the population with CHB, we recommend a multidisciplinary approach for disease management. This includes screening and treatment of metabolic comorbidities and providing lifestyle intervention programmes. Moreover, to prevent disease progression as a result of CHB, the role of early antiviral treatment in this population is up for debate. Whether regression of MAFLD, improvements in metabolic health, or early treatment is beneficial on liver-related outcomes in patients with CHB has yet to be determined.

Although this is one of the largest biopsy-controlled, multi-ethnic cohorts with patients with CHB to date, spanning over 20 years of follow-up, there are some limitations. First, this is a retrospective cohort study, and data on metabolic comorbidities were not systematically collected. Although we have excluded patients with steatosis with missing insufficient data for classification as MAFLD or no MAFLD, our approach might potentially have misclassified few lean patients without diabetes but with fatty liver disease as having no MAFLD if multiple minor metabolic dysfunctions were present but not assessed. However, such misclassification would not impact any of our findings, as it would have resulted in including at-risk patients in the control group, causing bias towards finding no difference in adverse event risk. Secondly, although the long duration of follow-up is an important strength of our cohort, it should be appreciated that patients without MAFLD at baseline might have developed this during follow-up. This issue would only have mitigated the observed differences in our cohort and is therefore unlikely to have had a significant impact on our findings. Furthermore, given the long follow-up duration, patients may have received various forms of antiviral therapy over time. Although we adjusted for having received antiviral therapy during follow-up, not all effects of antiviral therapy may be captured by these analyses. However, because patients with MAFLD had more often elevated ALT, they would have been managed more aggressively, making it unlikely that undertreatment of patients with concomitant fatty liver disease has influenced the results of our study. Next, diagnosis of steatohepatitis in patients with CHB is challenging. Current guidelines define NASH based on the presence of steatosis with (lobular) inflammation and ballooning. Because patients with MAFLD have steatosis by definition, only inflammatory activity and ballooning can be used for classification. In our group of patients with CHB and MAFLD and data on inflammatory patterns, 99% had lobular inflammation. Inflammatory activity therefore does not have significant discriminatory value in this context. This indicates that ballooning is the main discriminating factor in diagnosing steatohepatitis in patients with CHB and MAFLD. These limitations in defining steatohepatitis among patients with CHB may account for the absence of a significantly increased risk of adverse events for the pressence of steatohepatitis among patients with MAFLD. Finally, alcohol use is a well-recognised risk factor for liver disease progression. Patients with known alcoholic liver disease were excluded from this cohort, but a subset of our patients reported (previous) alcohol use. Adding (previous) alcohol use to our models did not influence any of the reported associations.

In conclusion, our study shows that MAFLD is independently associated with impaired event-free, HCC-free, and transplant-free survival in patients with CHB. Among patients with MAFLD, concomitant presence of steatohepatitis did not influence the risk of adverse outcomes. Our findings provide the first evidence for the clinical usefulness of the novel MAFLD criteria in CHB and highlight the importance of metabolic health in these patients.

## Financial support

Financial support was provided by the Foundation for Liver and Gastrointestinal Research, Rotterdam, The Netherlands. The funding source had no influence on study design, data collection, analysis and interpretation of the data, or the writing of the report and decision to submit the manuscript for publication.

## Authors’ contributions

Collection of data: LvK, HC, WPB, MJS. Study design, data analysis, and writing of the manuscript: LvK, WPB, HLAJ, RdK, MJS. Critical review of the manuscript and approval of the final version: LvK, HC, WPB, BH, KP, RdM, HLAJ, RdK, MJS. Approval of the submission of the manuscript: All authors.

## Data availability statement

Data collected for the study will not be made available for sharing.

## Conflicts of interest

MJS has received speaker's fees and research support from Fujirebio and has received grants from Gilead. KP consults for, advises, and received grants from Gilead. HLAJ received grants from Abbvie, Arbutus, Bristol Myers, Squibb, Gilead Sciences, Janssen, Merck, and Roche and is a consultant for Aligos, Arbutus, Arena, Eiger, Enyo, Gilead Sciences, GlaxoSmithKline, Janssen, Merck, Regulus, Roche, VBI Vaccines (Variation Biotechnologies), Vir Biotechnology Inc., and Viroclinics. RdK is a speaker for Echosens, consultant for AbbVie, and received grants from Abbvie, Gilead, and Janssen.

The remaining authors report no relevant conflicts.

Please refer to the accompanying ICMJE disclosure forms for further details.
