# Supplementary material for: Metabolic dysfunction-associated fatty liver disease increases risk of adverse outcomes in patients with chronic hepatitis B
Source: JHEP Rep. 2021 Aug 8;3(5):100350. doi: 10.1016/j.jhepr.2021.100350 (PMC8446794; doi:10.1016/j.jhepr.2021.100350)
Supplement: Multimedia component 1 [file mmc1.pdf]

Metabolic dysfunction-associated fatty liver disease increases risk of adverse outcomes in patients with chronic hepatitis B

Laurens A. van Kleef, Hannah S.J. Choi, Willem P. Brouwer, Bettina E. Hansen, Keyur Patel, Robert A. de Man, Harry L.A. Janssen, Robert J. de Knegt, Milan J. Sonneveld

Table of contents

Fig. S1.. 2

Table S1.. 3

Table S2.. 4

Fig. S1.

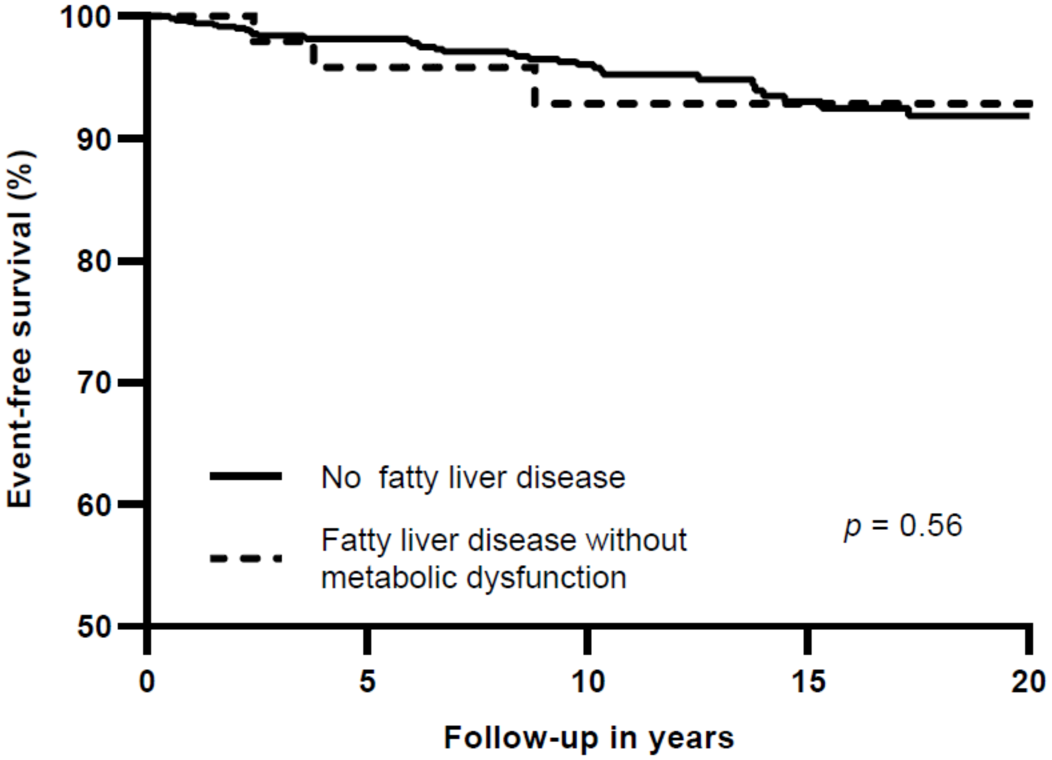

Fig. S1. Event-free survival in patients with fatty liver disease not complying MAFLD compared to patients without MAFLD.

MAFLD, metabolic dysfunction associated fatty liver disease

Table S1. Characteristics of patients with MAFLD according to presence of steatohepatitis

Variable	MAFLD without steatohepatitis n = 162	MAFLD with steatohepatitis n = 134	*p*
Age (years)	43.3 (11.2)	43.9 (12.1)	0.680
Female, n (%)	35 (21.6)	24 (17.9)	0.518
Race, n (%)			0.198
Caucasian	47 (29.0)	45 (33.6)	
Asian	99 (61.1)	69 (51.5)	
African/Black	12 (7.4)	18 (13.4)	
Other	4 (2.5)	2 (1.5)	
Overweight*, n (%)	154 (96.2)	125 (98.4)	0.453
Hypertension/hyperlipidemia, n (%)	37 (22.8)	39 (29.1)	0.274
Diabetes, n (%)	14 (8.6)	20 (14.9)	0.132
ALT (lU/L)	50 [36, 77]	56 [41, 82]	0.084
Elevated ALT†, n (%)	106 (69.7)	110 (82.7)	0.016
HBeAg-positive, n (%)	57 (35.2)	34 (25.4)	0.090
HBV DNA (log IU/mL)	5.15 (2.70)	4.40 (2.50)	0.016
Hepatic activity (A2-4), n (%)	71 (43.8)	82 (61.2)	0.004
Advanced fibrosis (F3-4), n (%)	46 (28.4)	48 (35.8)	0.215

Data is presented as mean (SD), median [P25-P75] or n and percentage. *BMI > 25 kg/m2 (non-Asians) or > 23 kg/m2 (Asians) †exceeding local ULN

Table S2. Prevalence of events for MAFLD and no MAFLD patients

**Variable**	**No MAFLD** n = 780	**MAFLD** n = 296
≥ 1 event	42 (5.4)	36 (12.2)
Decompensation	12 (1.5)	9 (3.0)
Hepatocellular carcinoma	18 (2.3)	18 (6.1)
Liver transplant	4 (0.5)	3 (1.0)
All-cause death	25 (3.2)	18 (6.1)

Data is presented as n and percentage.
