# Supplementary material for: Metabolic dysfunction-associated fatty liver disease increases risk of adverse outcomes in patients with chronic hepatitis B
Source: JHEP Rep. 2021 Aug 8;3(5):100350. doi: 10.1016/j.jhepr.2021.100350 (PMC8446794; doi:10.1016/j.jhepr.2021.100350)
Supplement: Multimedia component 3 [file mmc3.pdf]

ICMJE DISCLOSURE FORM

Date:______22-6-2021____
Your Name:_________R. J. de Knegt____
Manuscript Title: Metabolic dysfunction Associated Fatty Liver Disease increases risk for adverse outcomes in patients with Chronic Hepatitis B
Manuscript number (if known): JHEPR-D-21-00155

In the interest of transparency, we ask you to disclose all relationships/activities/interests listed below that are related to the content of your manuscript. "Related" means any relation with for-profit or not-for-profit third parties whose interests may be affected by the content of the manuscript. Disclosure represents a commitment to transparency and does not necessarily indicate a bias. If you are in doubt about whether to list a relationship/activity/interest, it is preferable that you do so.

The following questions apply to the author's relationships/activities/interests as they relate to the current manuscript only.

The author's relationships/activities/interests should be defined broadly. For example, if your manuscript pertains to the epidemiology of hypertension, you should declare all relationships with manufacturers of antihypertensive medication, even if that medication is not mentioned in the manuscript.

In item #1 below, report all support for the work reported in this manuscript without time limit. For all other items, the time frame for disclosure is the past 36 months.

		**Name all entities with whom you have this relationship or indicate none (add rows as needed)**	**Specifications/Comments (e.g., if payments were made to you or to your institution)**
		**Time frame: Since the initial planning of the work**	
1	All support for the present manuscript (e.g., funding, provision of study materials, medical writing, article processing charges, etc.) **No time limit for this item.**	__X__ None	
		**Time frame: past 36 months**	
2	Grants or contracts from any entity (if not indicated in item #1 above).	____ None	Abbvie, Gilead and Janssen
3	Royalties or licenses	__X__ None	
4	Consulting fees	____ None	Abbvie

5	Payment or honoraria for lectures, presentations, speakers bureaus, manuscript writing or educational events	____ None	EchoSens
6	Payment for expert testimony	__X__ None	
7	Support for attending meetings and/or travel	__X__ None	
8	Patents planned, issued or pending	__X__ None	
9	Participation on a Data Safety Monitoring Board or Advisory Board	__X__ None	
10	Leadership or fiduciary role in other board, society, committee or advocacy group, paid or unpaid	__X__ None	
11	Stock or stock options	__X__ None	
12	Receipt of equipment, materials, drugs, medical writing, gifts or other services	__X__ None	
13	Other financial or non-financial interests	__X__ None	

Please place an "X" next to the following statement to indicate your agreement:

__X_ I certify that I have answered every question and have not altered the wording of any of the questions on this form.

ICMJE DISCLOSURE FORM

Date:_______________________21-6-2021____
Your Name:______________Bettina E Hansen____
Manuscript Title: Metabolic dysfunction Associated Fatty Liver Disease increases risk for adverse outcomes in patients with Chronic Hepatitis B
Manuscript number (if known): JHEPR-D-21-00155

In the interest of transparency, we ask you to disclose all relationships/activities/interests listed below that are related to the content of your manuscript. "Related" means any relation with for-profit or not-for-profit third parties whose interests may be affected by the content of the manuscript. Disclosure represents a commitment to transparency and does not necessarily indicate a bias. If you are in doubt about whether to list a relationship/activity/interest, it is preferable that you do so.

The following questions apply to the author's relationships/activities/interests as they relate to the current manuscript only.

The author's relationships/activities/interests should be defined broadly. For example, if your manuscript pertains to the epidemiology of hypertension, you should declare all relationships with manufacturers of antihypertensive medication, even if that medication is not mentioned in the manuscript.

In item #1 below, report all support for the work reported in this manuscript without time limit. For all other items, the time frame for disclosure is the past 36 months.

		**Name all entities with whom you have this relationship or indicate none (add rows as needed)**	**Specifications/Comments (e.g., if payments were made to you or to your institution)**
		**Time frame: Since the initial planning of the work**	
1	All support for the present manuscript (e.g., funding, provision of study materials, medical writing, article processing charges, etc.) **No time limit for this item.**	____ None	
		**Time frame: past 36 months**	
2	Grants or contracts from any entity (if not indicated in item #1 above).	____ None	
3	Royalties or licenses	____ None	
4	Consulting fees	____ None	

5	Payment or honoraria for lectures, presentations, speakers bureaus, manuscript writing or educational events	_____ None	
6	Payment for expert testimony	_____ None	
7	Support for attending meetings and/or travel	_____ None	
8	Patents planned, issued or pending	_____ None	
9	Participation on a Data Safety Monitoring Board or Advisory Board	_____ None	
10	Leadership or fiduciary role in other board, society, committee or advocacy group, paid or unpaid	_____ None	
11	Stock or stock options	_____ None	
12	Receipt of equipment, materials, drugs, medical writing, gifts or other services	_____ None	
13	Other financial or non-financial interests	_____ None	

Please place an "X" next to the following statement to indicate your agreement:

_x__ I certify that I have answered every question and have not altered the wording of any of the questions on this form.

ICMJE DISCLOSURE FORM

Date:_____________________**25-06-2021**____
Your Name:__________________**HLA Janssen**____
Manuscript Title: Metabolic dysfunction Associated Fatty Liver Disease increases risk for adverse outcomes in patients with Chronic Hepatitis B
Manuscript number (if known): JHEPR-D-21-00155

In the interest of transparency, we ask you to disclose all relationships/activities/interests listed below that are related to the content of your manuscript. "Related" means any relation with for-profit or not-for-profit third parties whose interests may be affected by the content of the manuscript. Disclosure represents a commitment to transparency and does not necessarily indicate a bias. If you are in doubt about whether to list a relationship/activity/interest, it is preferable that you do so.

The following questions apply to the author's relationships/activities/interests as they relate to the current manuscript only.

The author's relationships/activities/interests should be defined broadly. For example, if your manuscript pertains to the epidemiology of hypertension, you should declare all relationships with manufacturers of antihypertensive medication, even if that medication is not mentioned in the manuscript.

In item #1 below, report all support for the work reported in this manuscript without time limit. For all other items, the time frame for disclosure is the past 36 months.

		**Name all entities with whom you have this relationship or indicate none (add rows as needed)**	**Specifications/Comments (e.g., if payments were made to you or to your institution)**
		**Time frame: Since the initial planning of the work**	
1	All support for the present manuscript (e.g., funding, provision of study materials, medical writing, article processing charges, etc.) **No time limit for this item.**	__X__ None	
		**Time frame: past 36 months**	
2	Grants or contracts from any entity (if not indicated in item #1 above).	____ None	Abbvie, Arbutus, Bristol Myers, Squibb, Gilead Sciences, Janssen, Merck, Roche
3	Royalties or licenses	__X__ None	
4	Consulting fees	____ None	Aligos, Arbutus, Arena, Eiger, Enyo, Gilead

			Sciences, GlaxoSmithKline, Janssen, Merck, Regulus, Roche, VBI Vaccines (Variation Biotechnologies), Vir Biotechnology Inc., Viroclinics.
5	Payment or honoraria for lectures, presentations, speakers bureaus, manuscript writing or educational events	__X__ None	
6	Payment for expert testimony	__X__ None	
7	Support for attending meetings and/or travel	__X__ None	
8	Patents planned, issued or pending	__X__ None	
9	Participation on a Data Safety Monitoring Board or Advisory Board	__X__ None	
10	Leadership or fiduciary role in other board, society, committee or advocacy group, paid or unpaid	__X__ None	
11	Stock or stock options	__X__ None	
12	Receipt of equipment, materials, drugs, medical writing, gifts or other services	__X__ None	
13	Other financial or non-financial interests	__X__ None	

Please place an "X" next to the following statement to indicate your agreement:

__X_ I certify that I have answered every question and have not altered the wording of any of the questions on this form.

ICMJE DISCLOSURE FORM

Date:_7th June2021____
Your Name:__Keyur Patel____
Manuscript Title: Metabolic dysfunction Associated Fatty Liver Disease increases risk for adverse outcomes in patients with Chronic Hepatitis B
Manuscript number (if known): JHEPR-D-21-00155

In the interest of transparency, we ask you to disclose all relationships/activities/interests listed below that are related to the content of your manuscript. "Related" means any relation with for-profit or not-for-profit third parties whose interests may be affected by the content of the manuscript. Disclosure represents a commitment to transparency and does not necessarily indicate a bias. If you are in doubt about whether to list a relationship/activity/interest, it is preferable that you do so.

The following questions apply to the author's relationships/activities/interests as they relate to the current manuscript only.

The author's relationships/activities/interests should be defined broadly. For example, if your manuscript pertains to the epidemiology of hypertension, you should declare all relationships with manufacturers of antihypertensive medication, even if that medication is not mentioned in the manuscript.

In item #1 below, report all support for the work reported in this manuscript without time limit. For all other items, the time frame for disclosure is the past 36 months.

		**Name all entities with whom you have this relationship or indicate none (add rows as needed)**	**Specifications/Comments (e.g., if payments were made to you or to your institution)**
		**Time frame: Since the initial planning of the work**	
1	All support for the present manuscript (e.g., funding, provision of study materials, medical writing, article processing charges, etc.) **No time limit for this item.**	__X__ None	
		**Time frame: past 36 months**	
2	Grants or contracts from any entity (if not indicated in item #1 above).	____ None	Gilead Sciences
3	Royalties or licenses	_X___ None	
4	Consulting fees	__X__ None	

5	Payment or honoraria for lectures, presentations, speakers bureaus, manuscript writing or educational events	____ None	Gilead Sciences
6	Payment for expert testimony	_X___ None	
7	Support for attending meetings and/or travel	__X__ None	
8	Patents planned, issued or pending	__X__ None	
9	Participation on a Data Safety Monitoring Board or Advisory Board	____ None	Gilead Sciences
10	Leadership or fiduciary role in other board, society, committee or advocacy group, paid or unpaid	__X__ None	
11	Stock or stock options	__X__ None	
12	Receipt of equipment, materials, drugs, medical writing, gifts or other services	__X__ None	
13	Other financial or non-financial interests	_X___ None	

Please place an "X" next to the following statement to indicate your agreement:

_X__ I certify that I have answered every question and have not altered the wording of any of the questions on this form.

ICMJE DISCLOSURE FORM

Date:________21-6-2021____
Your Name:____L.A. van Kleef____
Manuscript Title: Metabolic dysfunction Associated Fatty Liver Disease increases risk for adverse outcomes in patients with Chronic Hepatitis B
Manuscript number (if known): JHEPR-D-21-00155

In the interest of transparency, we ask you to disclose all relationships/activities/interests listed below that are related to the content of your manuscript. "Related" means any relation with for-profit or not-for-profit third parties whose interests may be affected by the content of the manuscript. Disclosure represents a commitment to transparency and does not necessarily indicate a bias. If you are in doubt about whether to list a relationship/activity/interest, it is preferable that you do so.

The following questions apply to the author's relationships/activities/interests as they relate to the current manuscript only.

The author's relationships/activities/interests should be defined broadly. For example, if your manuscript pertains to the epidemiology of hypertension, you should declare all relationships with manufacturers of antihypertensive medication, even if that medication is not mentioned in the manuscript.

In item #1 below, report all support for the work reported in this manuscript without time limit. For all other items, the time frame for disclosure is the past 36 months.

		**Name all entities with whom you have this relationship or indicate none (add rows as needed)**	**Specifications/Comments (e.g., if payments were made to you or to your institution)**
		**Time frame: Since the initial planning of the work**	
1	All support for the present manuscript (e.g., funding, provision of study materials, medical writing, article processing charges, etc.) **No time limit for this item.**	__X__ None	
		**Time frame: past 36 months**	
2	Grants or contracts from any entity (if not indicated in item #1 above).	__X__ None	
3	Royalties or licenses	__X__ None	
4	Consulting fees	__X__ None	

5	Payment or honoraria for lectures, presentations, speakers bureaus, manuscript writing or educational events	__X__ None	
6	Payment for expert testimony	__X__ None	
7	Support for attending meetings and/or travel	__X__ None	
8	Patents planned, issued or pending	__X__ None	
9	Participation on a Data Safety Monitoring Board or Advisory Board	__X__ None	
10	Leadership or fiduciary role in other board, society, committee or advocacy group, paid or unpaid	__X__ None	
11	Stock or stock options	__X__ None	
12	Receipt of equipment, materials, drugs, medical writing, gifts or other services	__X__ None	
13	Other financial or non-financial interests	__X__ None	

Please place an "X" next to the following statement to indicate your agreement:

__X_ I certify that I have answered every question and have not altered the wording of any of the questions on this form.

ICMJE DISCLOSURE FORM

Date:_23-06-2021____
Your Name: MJ Sonneveld____
Manuscript Title: Metabolic dysfunction Associated Fatty Liver Disease increases risk for adverse outcomes in patients with Chronic Hepatitis B
Manuscript number (if known): JHEPR-D-21-00155

In the interest of transparency, we ask you to disclose all relationships/activities/interests listed below that are related to the content of your manuscript. "Related" means any relation with for-profit or not-for-profit third parties whose interests may be affected by the content of the manuscript. Disclosure represents a commitment to transparency and does not necessarily indicate a bias. If you are in doubt about whether to list a relationship/activity/interest, it is preferable that you do so.

The following questions apply to the author's relationships/activities/interests as they relate to the current manuscript only.

The author's relationships/activities/interests should be defined broadly. For example, if your manuscript pertains to the epidemiology of hypertension, you should declare all relationships with manufacturers of antihypertensive medication, even if that medication is not mentioned in the manuscript.

In item #1 below, report all support for the work reported in this manuscript without time limit. For all other items, the time frame for disclosure is the past 36 months.

		Name all entities with whom you have this relationship or indicate none (add rows as needed)	Specifications/Comments (e.g., if payments were made to you or to your institution)
**Time frame: Since the initial planning of the work**			
1	All support for the present manuscript (e.g., funding, provision of study materials, medical writing, article processing charges, etc.) **No time limit for this item.**	____ None	
**Time frame: past 36 months**			
2	Grants or contracts from any entity (if not indicated in item #1 above).	____ Gilead, Fujirebio	
3	Royalties or licenses	____ None	
4	Consulting fees	____ Fujirebio	

5	Payment or honoraria for lectures, presentations, speakers bureaus, manuscript writing or educational events	____ None	
6	Payment for expert testimony	____ None	
7	Support for attending meetings and/or travel	____ None	
8	Patents planned, issued or pending	____ None	
9	Participation on a Data Safety Monitoring Board or Advisory Board	____ None	
10	Leadership or fiduciary role in other board, society, committee or advocacy group, paid or unpaid	____ None	
11	Stock or stock options	____ None	
12	Receipt of equipment, materials, drugs, medical writing, gifts or other services	____ fujirebio	
13	Other financial or non-financial interests	____ None	

Please place an "X" next to the following statement to indicate your agreement:

__X_ I certify that I have answered every question and have not altered the wording of any of the questions on this form.

ICMJE DISCLOSURE FORM

Date___07-06-2021____
Your Name:_Robert A. de Man____
Manuscript Title: Metabolic dysfunction Associated Fatty Liver Disease increases risk for adverse outcomes in patients with Chronic Hepatitis B
Manuscript number (if known): JHEPR-D-21-00155

In the interest of transparency, we ask you to disclose all relationships/activities/interests listed below that are related to the content of your manuscript. "Related" means any relation with for-profit or not-for-profit third parties whose interests may be affected by the content of the manuscript. Disclosure represents a commitment to transparency and does not necessarily indicate a bias. If you are in doubt about whether to list a relationship/activity/interest, it is preferable that you do so.

The following questions apply to the author's relationships/activities/interests as they relate to the current manuscript only.

The author's relationships/activities/interests should be defined broadly. For example, if your manuscript pertains to the epidemiology of hypertension, you should declare all relationships with manufacturers of antihypertensive medication, even if that medication is not mentioned in the manuscript.

In item #1 below, report all support for the work reported in this manuscript without time limit. For all other items, the time frame for disclosure is the past 36 months.

		**Name all entities with whom you have this relationship or indicate none (add rows as needed)**	**Specifications/Comments (e.g., if payments were made to you or to your institution)**
		**Time frame: Since the initial planning of the work**	
1	All support for the present manuscript (e.g., funding, provision of study materials, medical writing, article processing charges, etc.) **No time limit for this item.**	____ None	
		**Time frame: past 36 months**	
2	Grants or contracts from any entity (if not indicated in item #1 above).	____ None	
3	Royalties or licenses	____ None	
4	Consulting fees	____ None	

5	Payment or honoraria for lectures, presentations, speakers bureaus, manuscript writing or educational events	____ None	
6	Payment for expert testimony	____ None	
7	Support for attending meetings and/or travel	____ None	
8	Patents planned, issued or pending	____ None	
9	Participation on a Data Safety Monitoring Board or Advisory Board	____ None	
10	Leadership or fiduciary role in other board, society, committee or advocacy group, paid or unpaid	____ None	
11	Stock or stock options	____ None	
12	Receipt of equipment, materials, drugs, medical writing, gifts or other services	____ None	
13	Other financial or non-financial interests	____ None	

Please place an "X" next to the following statement to indicate your agreement:

__x_ I certify that I have answered every question and have not altered the wording of any of the questions on this form.

ICMJE DISCLOSURE FORM

Date:__11-06-2021____
Your Name:__WP Brouwer____
Manuscript Title: Metabolic dysfunction Associated Fatty Liver Disease increases risk for adverse outcomes in patients with Chronic Hepatitis B
Manuscript number (if known): JHEPR-D-21-00155

In the interest of transparency, we ask you to disclose all relationships/activities/interests listed below that are related to the content of your manuscript. "Related" means any relation with for-profit or not-for-profit third parties whose interests may be affected by the content of the manuscript. Disclosure represents a commitment to transparency and does not necessarily indicate a bias. If you are in doubt about whether to list a relationship/activity/interest, it is preferable that you do so.

The following questions apply to the author's relationships/activities/interests as they relate to the current manuscript only.

The author's relationships/activities/interests should be defined broadly. For example, if your manuscript pertains to the epidemiology of hypertension, you should declare all relationships with manufacturers of antihypertensive medication, even if that medication is not mentioned in the manuscript.

In item #1 below, report all support for the work reported in this manuscript without time limit. For all other items, the time frame for disclosure is the past 36 months.

		**Name all entities with whom you have this relationship or indicate none (add rows as needed)**	**Specifications/Comments (e.g., if payments were made to you or to your institution)**
		**Time frame: Since the initial planning of the work**	
1	All support for the present manuscript (e.g., funding, provision of study materials, medical writing, article processing charges, etc.) **No time limit for this item.**	__X__ None	
		**Time frame: past 36 months**	
2	Grants or contracts from any entity (if not indicated in item #1 above).	__X__ None	
3	Royalties or licenses	__X__ None	
4	Consulting fees	_X___ None	

5	Payment or honoraria for lectures, presentations, speakers bureaus, manuscript writing or educational events	___X_ None	
6	Payment for expert testimony	__X__ None	
7	Support for attending meetings and/or travel	___X_ None	
8	Patents planned, issued or pending	___X_ None	
9	Participation on a Data Safety Monitoring Board or Advisory Board	_X___ None	
10	Leadership or fiduciary role in other board, society, committee or advocacy group, paid or unpaid	__X__ None	
11	Stock or stock options	__X__ None	
12	Receipt of equipment, materials, drugs, medical writing, gifts or other services	__X__ None	
13	Other financial or non-financial interests	__X__ None	

Please place an "X" next to the following statement to indicate your agreement:

X I certify that I have answered every question and have not altered the wording of any of the questions on this form.

ICMJE DISCLOSURE FORM

Date: Mon, Jun 7, 2021
Your Name: HANNAH S.J. CHOI
Manuscript Title: Metabolic dysfunction Associated Fatty Liver Disease increases risk for adverse outcomes in patients with Chronic Hepatitis B
Manuscript number (if known): JHEPR-D-21-00155

In the interest of transparency, we ask you to disclose all relationships/activities/interests listed below that are related to the content of your manuscript. "Related" means any relation with for-profit or not-for-profit third parties whose interests may be affected by the content of the manuscript. Disclosure represents a commitment to transparency and does not necessarily indicate a bias. If you are in doubt about whether to list a relationship/activity/interest, it is preferable that you do so.

The following questions apply to the author's relationships/activities/interests as they relate to the current manuscript only.

The author's relationships/activities/interests should be defined broadly. For example, if your manuscript pertains to the epidemiology of hypertension, you should declare all relationships with manufacturers of antihypertensive medication, even if that medication is not mentioned in the manuscript.

In item #1 below, report all support for the work reported in this manuscript without time limit. For all other items, the time frame for disclosure is the past 36 months.

		**Name all entities with whom you have this relationship or indicate none (add rows as needed)**	**Specifications/Comments (e.g., if payments were made to you or to your institution)**
**Time frame: Since the initial planning of the work**			
1	All support for the present manuscript (e.g., funding, provision of study materials, medical writing, article processing charges, etc.) **No time limit for this item.**	✓ None	
**Time frame: past 36 months**			
2	Grants or contracts from any entity (if not indicated in item #1 above).	✓ None	
3	Royalties or licenses	✓ None	
4	Consulting fees	✓ None	

5	Payment or honoraria for lectures, presentations, speakers bureaus, manuscript writing or educational events	✓ None	
6	Payment for expert testimony	✓ None	
7	Support for attending meetings and/or travel	✓ None	
8	Patents planned, issued or pending	✓ None	
9	Participation on a Data Safety Monitoring Board or Advisory Board	✓ None	
10	Leadership or fiduciary role in other board, society, committee or advocacy group, paid or unpaid	✓ None	
11	Stock or stock options	✓ None	
12	Receipt of equipment, materials, drugs, medical writing, gifts or other services	✓ None	
13	Other financial or non-financial interests	✓ None	

Please place an "X" next to the following statement to indicate your agreement:

X **I certify that I have answered every question and have not altered the wording of any of the questions on this form.**
